# Genetic variability in sodium-glucose cotransporter 2 influences glycemic control and risk for diabetic retinopathy in type 2 diabetes patients

**DOI:** 10.2478/jomb-2019-0040

**Published:** 2020-09-02

**Authors:** Jasna Klen, Katja Goričar, Vita Dolžan

**Affiliations:** 1 General Hospital Trbovlje, Trbovlje, Slovenia; 2 University of Ljubljana, Faculty of Medicine, Institute of Biochemistry, Pharmacogenetics Laboratory, Ljubljana, Slovenia

**Keywords:** diabetes type 2, SLC5A2, SGLT2, genetic polymorphism, retinopathy, dijabetes tip 2, SLC5A2, SGLT2, genetički polimorfizam, retinopatija

## Abstract

**Background:**

Gluconeogenesis and renal glucose excretion in kidneys both play an important role in glucose homeostasis. Sodium-glucose cotransporter (SGLT2), coded by the SLC5A2 gene is responsible for reabsorption up to 99% of the filtered glucose in proximal tubules. SLC5A2 genetic polymorphisms were suggested to influence glucose homeostasis. We investigated if common SLC5A2 rs9934336 polymorphism influences glycemic control and risk for macro or microvascular complications in Slovenian type 2 diabetes (T2D) patients.

**Methods:**

All 181 clinically well characterized T2D patients were genotyped for SLC5A2 rs9934336 G>A polymorphism. Associations with glycemic control and T2D complications were assessed with nonparametric tests and logistic regression.

**Results:**

SLC5A2 rs9934336 was significantly associated with increased fasting blood glucose levels (P<0.001) and HbA1c levels under the dominant genetic model (P=0.030). After adjustment for T2D duration, significantly higher risk for diabetic retinopathy was present in carriers of at least one polymorphic SLC5A2 rs9934336 A allele compared to non-carriers (OR=7.62; 95%CI=1.65-35.28; P=0.009).

**Conclusions:**

Our pilot study suggests an important role of SLC5A2 polymorphisms in the physiologic process of glucose reabsorption in kidneys in T2D patients. This is also the first report on the association between SLC5A2 polymorphism and diabetic retinopathy.

## Abstract (sr)

**Uvod:**

Glukoneogeneza i izlučivanje glukoze u bubrezima imaju važnu ulogu u homeostazi glukoze. Natrijum-glukozni kotransporter (SGLT2), kodiran od strane gena SLC5A2, odgovoran je za reapsorpciju do 99% filtrirane glukoze u proksimalnim tubulima. Predloženo je da genetski polimorfizmi SLC5A2 utiču na homeostazu glukoze. Ispitali smo, da li učestali polimorfizam SLC5A2 rs9934336 utiče na glikemičku kontrolu i na rizik za makro i mikrovaskularne komplikacije kod pacijenata iz Slovenije sa dijabetesom tipa 2 (T2D).

**Metode:**

Svih 181 klinički dobro okarakterisanih pacijenata sa T2D je genotipizirano na polimorfizam SLC5A2 rs9934336 G>A. Povezanost sa kontrolom glikemije i komplikacijama T2D ispitivana je neparametrijskim testovima i logističkom regresijom.

**Rezultati:**

SLC5A2 rs9934336 je značajno povezan sa povećanim nivoima glukoze u krvi natašte (P <0,001) i nivoima HbA1c pod dominantnim genetskim modelom (P = 0,030). Nakon uzimanja u obzir trajanje T2D, značajno veci rizik za dijabetičku retinopatiju bio je prisutan kod nosilaca najmanje jednog polimorfnog alela SLC5A2 rs9934336 A u odnosu na pacijente koji nisu nosioci (OR = 7,62; 95% CI = 1,65-35,28; P = 0,009).

**Zaključak:**

Naša pilot-studija ukazuje na važnu ulogu polimorfizama SLC5A2 u fiziološkom procesu reapsorpcije glukoze u bubrezima kod pacijenata sa T2D. Ovo je tako|e prva studija o povezanosti polimorfizma SLC5A2 i dijabetičke retinopatije.

## Introduction

Type 2 diabetes (T2D) results from defects in insulin secretion, deficiency in insulin signaling and/or insulin resistance that may contribute to chronic hyperglycemia [1]. Micro and macrovascular complications are common long term complications of T2D that may be prevented or at least delayed with rapid diagnosis and proper antihyperglycemic therapy [2].

Kidneys have an important role in lowering glucose plasma levels as they filter around 180 liters of plasma every day, first by secreting approximately 160-180 grams of glucose per day and then by reabsorbing virtually all this glucose in normoglycemic subjects. Two sodium-glucose transporters (SGLT) belonging to the solute carrier family 5 (SLC5) play an important role in glucose reabsorption in the kidney. From the renal ultrafiltrate approximately 90% of glucose is reabsorbed in insulin independent process by SGLT2 (SLC5A2) in segments 1 and 2, while 10% is reabsorbed by SGLT1 in segment 3 (SLC5A1) of the proximal tubule [3]. As these transporters have a transport maximum for glucose reabsorption (TmG) from glomerular filtrate, maximal glucose reabsorption capacity (around 10 mmol/L) may be exceeded in T2D patients with hyperglycemia. The excess glucose which cannot be reabsorbed is excreted in the urine, leading to glucosuria [4] [5].

The increased capacity of the kidney to reabsorb glucose and increased TmG occur due to increased expression of SGLT2 in T2D patients with chronic hyperglycemia and contribute to the maintenance of hyperglycemia [5] [6]. SGLT2 inhibition has been recognized as a novel and safe approach to lowering high glucose levels in T2D and several SGLT2 inhibitors were developed and tested in clinical trials [7] [8]. In addition to their excellent high blood glucose lowering potential without the risk of hypoglycemias, they also showed both cardioprotective [7] and renoprotective effect [9] [10]. Further studies are needed to demonstrate if SGLT2 inhibitors have a protective effect against other late complications of T2D.

Several studies, including our own have previously shown that genetic factors play an important role in the risk of development late T2D complications [11] [12]. Genetic variability was also identified in the *SLC5A2* gene coding for SGLT2. Severe, but rare *SLC5A2* mutations were associated with familial renal glucosuria, a benign condition with normal blood glucose [13]. Initial studies of common functional polymorphisms identified in SLC5A2 suggested their influence on glucose homeostasis in non-diabetic subjects [14] however a recent study on T2D patients observed no significant effect of SLC5A2 genetic variability on patients' metabolic traits or their response to treatment with empagliflozin [15].

The aim of our study was to investigate if common polymorphism *SLC5A2* rs9934336 influences blood glucose levels and risk for macro and microvascular complications in Slovenian Type 2 diabetes patients even in the absence of treatment with SGLT2 inhibitors.

## Materials and Methods

### Patients

Our retrospective study included T2D patients aged between 18 to 75 years when they came for regular visits at the outpatient clinic at General Hospital Trbovlje. Most of the patients were treated with sulphonylurea either in monotherapy or in combination with metformin. Patients with end-stage renal failure due to diabetic nephropathy were mostly treated with insulin. Exclusion criteria were diabetes type 1, gestational diabetes, other types of diabetes, active cancer, heart failure New York Heart Association (NYHA) 3-4, co-treatment with corticosteroids or estrogens, conditions that can cause hyperglycaemia, addiction to alcohol or illegal drugs and dementia or severe psychiatric disorders as described in detail in our previous study [11]. Information on the history of diabetes, presence of arterial hypertension, hyperlipidemia and chronic diabetic complications, smoking status and other medications was obtained from the medical records. At least once per year plasma lipid levels, urea and creatinine and urine albumin and albumin/creatinine ratio were determined. Kidney function was assessed in line with the revised chronic kidney disease classification [16]. Albuminuria was classified as normal (<20 mg/L), moderately increased albuminuria (20-200 mg/L, formerly known as microalbuminuria) and severely increased albuminuria (>200 mg/L, formerly known as macroalbuminuria). Estimated glomerular filtration was calculated according to the Modification of Diet in Renal Disease Study (MDRD) [17]. Once per year patients were referred to consulting ophthalmologist for screening for diabetic retinopathy. Echosonography and exercise stress test (cycloergometry) were performed at the first visit and also at any complaints suggestive for ischemic heart disease.

The study was approved by the National Ethics Committee and conducted in accordance with the Declaration of Helsinki. Written informed consent was obtained from all subjects.

### SNP selection, DNA isolation and genotyping

*SLC5A2* rs9934336 (c.127-121G>A, intronic single nucleotide polymorphism (SNP)) was selected based on previously published literature [14]. We also checked for other common putatively functional nonintronic SNPs in *SLC5A2* using SNP database [18] but no other candidate SNP was found.

Genomic DNA was extracted from whole-blood frozen samples collected at the inclusion in the study using the Qiagen FlexiGene Kit (Qiagen, Hilden, Germany) according to the manufacturer's instructions.

Genotyping of *SLC5A2* rs9934336 was carried out using a fluorescence-based competitive allelespecific (KASPar) assay according to the manufacturer's instructions (LGC Genomics, UK). Genotyping was performed blind to any clinical data and was randomly repeated in 15% of samples. Genotyping quality control criteria were 100% duplicate call rate and 95% SNP-wise call rate. Duplicate call rate and SNPwise call rate were 100%.

### Statistical analysis

Median with interquartile range and frequencies were used to describe continuous and categorical variables, respectively. Chi-square test was used to assess potential deviation from Hardy-Weinberg equilibrium. Nonparametric Kruskal-Wallis and Mann-Whitney tests were used to evaluate the association with basal glucose and HbA1c, while logistic regression was used to assess risk for macro and microvascular complications. All statistical analyses were performed using IBM SPSS Statistics 19.0 (IBM Corporation, Armonk, NY, USA). All statistical tests were two sided. To lower the chance of false positive findings, Benjamini-Hochberg false discovery rate was used to account for multiple comparisons [19]. The level of statistical significance was set at 0.004 and P values between 0.004 and 0.050 were considered as nominally significant.

Based on measurements from previous studies, we could detect differences between groups of ±0.42% for glycated hemoglobin (HbA1c) or ±0.85 mmol/l for fasting glucose with 80% power based on our sample size. For macrovascular complications, based on our sample size and *SLC5A2* rs9934336 minor allele frequency, we could detect odds ratios 0.23 or 2.69 with 80% power. For microvascular complications, based on our sample size and *SLC5A2* rs9934336 minor allele frequency, we could detect odds ratios 0.18 or 2.97 with 80% power.

## Results

### Patients' characteristics

Our study included 181 T2D patients, 105 (58.0%) male and 76 (42%) female, with median age 64 (58.5 -70.5) years. Median T2D duration at the inclusion in the study was 11 (6–17) years.

Most of the patients were treated with sulphonylurea, either in monotherapy (21; 13.5%) or in combination with metformin (135; 86.5%). Among 25 hemodialysis patients with end-stage renal failure due to diabetic nephropathy, 20 (80%) were on insulin treatment, 1 (4%) patient was treated with sulphonylurea in monotherapy, while others (16%) were on diet. Fasting glucose levels were available for 104 patients, and 94 (90.4%) had values above 6.1 mmol/L. Median fasting glucose level was 7.5 (6.7-8.7) mmol/L. Still, most of the patients had relatively normal blood glucose control with median HbA1c 6.8% (6.3–7.6). Patients also had well controlled blood pressure and plasma lipid levels (Table 1). No significant differences were observed with regards to the laboratory parameters between males and females, except for the slightly higher HDL-cholesterol levels in females (P=0.008) (data not shown). Regarding kidney function, 51 patients had stage 1, 105 patients had stage 2 or 3 chronic kidney disease, and 25 patients had end-stage (stage 5) renal failure due to diabetic nephropathy and required hemodialysis.

**Table 1:** Patients characteristics Data are shown as median (25%–75%), except for: ^a^N (%).^b^Data available for 104 patients.

	T2D patients (N=181)
Male gender^a^	105 (58.0)
Age (years)	64.0 (58.5–70.5)
Duration of T2D (years)	11.0 (6.0–17.0)
HbA1c (%), mmol/mol	6.9 (6.3–7.6) [52 (45–60)]
Fasting blood glucose (mmol/L)^b^	7.5 (6.7–8.7)
BMI (kg/m^2^)	30.0 (28.0–33.3)
Blood pressure systolic (mmHg)	135.0 (130.0–145.0)
Blood pressure diastolic (mmHg)	80.0 (70.0–80.0)
Total cholesterol (mmol/L)	4.2 (3.5–5.0)
LDL-cholesterol (mmol/L)	2.4 (1.9–3.1)
HDL-cholesterol (mmol/L)	1.1 (1.0–1.4)
TAG (mmol/L)	1.6 (1.2–2.4)

### Genotyping analysis

*SLC5A2* rs9934336 genotype distribution was as follows: 101 (55.8%) of patients were homozygous for normal GG genotype, 67 (37.0%) were GA heterozygous and 13 (7.2%) were homozygous for polymorphic AA genotype. Minor allele frequency was 0.257. The distributions was in agreement with Hardy-Weinberg equilibrium (P=0.682). Genotype frequencies were in HWE also in all subgroups with late complications (data not shown).

### Association with fasting glucose and HbA1c levels

**Figure 1:**
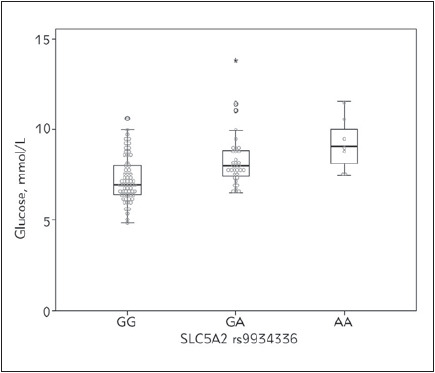
Fasting blood glucose levels in T2D patients according to *SLC5A2* rs9934336 genotype

*SLC5A2* rs9934336 was significantly associated with increased fasting blood glucose levels both in additive and dominant genetic models (both P<0.001). Fasting glucose levels were 6.96 (6.40-7.99) mmol/L for patients with normal GG genotype, 7.96 (7.31-8.83) mmol/L for patients with GA genotype and 9.04 (7.43-10.51) mmol/L for polymorphic AA genotype (Figure 1). The association of rs9934336 genotype with slightly higher HbA1c levels was nominally significant only under the dominant genetic model (P=0.030) (Table 2).

**Table 2:** The association of *SLC5A2* rs9934336 genotype with fasting glucose and HbA1c levels in T2D patients ^a^data available for 104 patients, ^b^comparison using Kruskal-Wallis test, ^c^comparison using Mann-Whitney test

Genotype	Fasting glucose (mmol/L)^a^	HbA1c (%)
	Median (25% –75%)	Median (25% –75%)
GG	6.96 (6.40–7.99)	6.7 (6.3–7.3)
GA	7.96 (7.31–8.83)	6.9 (6.4–7.7)
AA	9.04 (7.43–10.51)	7.1 (6.7–8.2)
P^b^	<0.001	0.079
GA+AA	8.04 (7.43–8.92)	7.0 (6.4–7.7)
P^c ^	<0.001	0.030

### Late complications

Macrovascular complications were observed in 46 (25.4%) patients: 10 had peripheral arterial occlusive disease (PAOD), 16 ischemic cerebral disease (ICD) and 32 myocardial infarction (MI). Microvascular complications were observed in 34 (18.8%) patients: 25 (13.8%) patients had end stage kidney failure due to diabetic nephropathy, 13 (7.2%) neuropathy and 15 (8.3%) retinopathy.

HbA1c was not significantly associated with risk for macrovascular complications (OR=0.75; 95% CI=0.53-1.06; P=0.102), but tended to be associated with slightly decreased risk for microvascular complications (OR=0.67; 95% CI=0.45-0.99; P=0.042). In the subgroup with available fasting blood glucose data, median blood glucose level was not significantly associated with risk for macrovascular complications (OR=0.81; 95% CI=0.56 -1.18; P=0.276) or microvascular complications (OR=0.57; 95% CI=0.26 -1.22; P=0.147).

In logistic regression analysis, longer duration of T2D and lower HDL cholesterol levels were the most important clinical risk factors increasing the odds for macrovascular complications (OR=1.08; 95% CI= 1.03-1.12; P=0.001 and OR=0.20; 95% CI= 0.06-0.64; P=0.007, respectively). The most important clinical risk factor increasing the odds for microvascular complications was longer duration of T2D (OR=1.12; 95% CI=1.07-1.17; P < 0.001). These parameters were therefore used in for adjustment in multivariable logistic regression.

Retinopathy was the only late T2D complication associated with *SLC5A2* polymorphism after adjustment for T2D duration (Table 3). Carriers of at least one polymorphic *SLC5A2* rs9934336 A allele had nominally significantly higher risk for diabetic retinopathy than non-carriers (OR=7.62; 95%CI=1.65-35.28; P=0.009).

**Table 3:** The association of *SLC5A2* rs9934336 with the risk for macrovascular and microvascular complications in type 2 diabetes patients (adjusted for clinical parameters) P values are adjusted for T2D duration and HDL cholesterol for macrovascular complications and adjusted for T2D duration for microvascular complications. CI, confidence interval; ICD, ischemic cerebral disease; MI, myocardial infarction; N, number of patients; OR, odds ratio; PAOD, peripheral arterial occlusive disease.

Complication		Genotype			
		GG	GA	AA	GA+AA
Macrovascular	N (%)	28 (27.7)	14 (20.9)	4 (30.8)	18 (22.5)
	OR (95 % CI)	Reference	0.71 (0.32–1.55)	0.95 (0.26–3.51)	0.75 (0.36–1.55)
	P		0.384	0.934	0.436
PAOD	N (%)	6 (5.9)	4 (6.0)	0 (0.0)	4 (5.0)
	OR (95 % CI)	Reference	1.01 (0.26–3.89)	/	0.81 (0.21–3.07)
	P		0.987	/	0.752
ICD	N (%)	9 (8.9)	5 (7.5)	2 (15.4)	7 (8.8)
	OR (95 % CI)	Reference	0.87 (0.27–2.78)	1.65 (0.31–8.87)	1.01 (0.35–2.90)
	P		0.811	0.557	0.993
MI	N (%)	20 (19.8)	10 (14.9)	2 (15.4)	12 (15.0)
	OR (95 % CI)	Reference	0.78 (0.32–1.89)	0.63 (0.12–3.31)	0.75 (0.33–1.73)
	P		0.579	0.587	0.500
Microvascular	N (%)	19 (18.8)	13 (19.4)	2 (15.4)	15 (18.8)
	OR (95 % CI)	Reference	1.25 (0.53–2.95)	0.75 (0.14–4.05)	1.15 (0.51–2.61)
	P		0.606	0.741	0.733
End-stage kidney	N (%)	14 (13.9)	9 (13.4)	2 (15.4)	11 (13.8)
	OR (95 % CI)	Reference	1.24 (0.45–3.40)	1.21 (0.21–7.04)	1.23 (0.47–3.20)
	P		0.677	0.833	0.666
Retinopathy	N (%)	5 (5.0)	8 (11.9)	2 (15.4)	10 (12.5)
	OR (95 % CI)	Reference	7.39 (1.53–35.65)	8.83 (0.92–84.57)	7.62 (1.65–35.28)
	P		0.013	0.059	0.009
Neuropathy	N (%)	6 (5.9)	5 (7.6)	2 (15.4)	7 (8.9)
	OR (95 % CI)	Reference	1.56 (0.44–5.58)	3.06 (0.52-17.90)	1.82 (0.56–5.90)
	P		0.491	0.214	0.317

## Discussion

In the present study we investigated the role of genetic variability in the principal glucose transporter SGLT2 on glycaemia and long term outcomes in T2D patients not treated with SGLT2 inhibitors. We have shown that genetic variability of *SLC5A2* gene coding for SGLT2 influences both fasting glucose levels and HbA1c. In addition we have observed an association of *SLC5A2* rs9934336 polymorphism with the risk for diabetic retinopathy, although no associations were observed with other microvascular or macrovascular late complications of T2D.

The most important finding of our study was that *SLC5A2* rs9934336 polymorphism significantly influenced fasting blood glucose levels in T2D patients. We even observed a gene dose effect, with the carriers of one polymorphic *SLC5A2* rs9934336 A allele having higher median fasting glucose levels than non-carriers, while carriers of two polymorphic had the highest fasting blood glucose levels. The effect of *SLC5A2* rs9934336 genotypes on HbA1c levels was less pronounced, however significant under the dominant genetic model. Modest association of *SLC5A2* rs9934336 polymorphism with glucose concentrations during oral glucose tolerance test was also observed in a cohort of German Sorb nondiabetic individuals with either normal glucose tolerance, impaired fasting glucose or impaired glucose tolerance. However in this particular group, carriers of polymorphic rs9934336 AA genotype had reduced glucose concentrations 30-min after oral glucose load [14]. Another German study showed no association of this polymorphism with fasting plasma glucose levels, HbA1c or the glucose concentrations during oral glucose tolerance test neither in individuals with increased risk for T2D nor in T2D patients treated with empagliflozin [15]. Regarding the small number of studies and the discrepant results, the role of *SLC5A2* rs9934336 polymorphism in glucose homeostasis remains to be determined. Furthermore, functional studies should be performed to elucidate the role of this polymorphism in glucose transport.

To our knowledge, so far only one study reported that common genetic variants in the SLC5A2 gene did not affect diabetes-related metabolic traits such as body fat or systolic blood pressure which may represent a risk for development of late T2D complications [15]. However in our study we observed an interesting association of *SLC5A2* rs9934336 poly morphism and the risk for development of diabetic retinopathy, but no other associations with either other microvascular or macrovascular late complications of T2D.

Diabetic retinopathy is most common late complication of T2D that significantly decreases patient's quality of life. Although the mechanisms underlying these conditions have been extensively studied, they remain unknown [20] [21]. Animal model studies showed that in addition to expression in renal proximal tubular cells and other tissues [22] SGLT2 may be also expressed in bovine retinal pericytes [23]. Among other important physiological functions, pericytes in the retina participate in microcirculation control and microvessel protection [23].

Pericytes play an important role in the development of diabetic retinopathy as pericyte swelling and loss was shown to occur in the early stage of diabetic retinopathy and leads to microaneurysm formation in the retina [24] [25].

It has been suggested that SGLT2 in retinal pericytes may have role of glucose sensor that controls cellular tone in response to changes in extracellular glucose concentrations [26]. These findings may support the biological plausibility of our observations that genetic variability of SGLT2 may play a role in the development of diabetic retinopathy. It has been also show that glucose entry into pericytes increased twofold under hyperglycemia conditions [26]. Such alterations in glucose supply could potentially change retinal energy metabolism and therefor result in complications [27].

Excessive SGLT2 mediated entry of glucose and sodium during high glucose conditions resulted in functional and morphological changes in retinal peri-cytes, but this effect was attenuated by nonselective SGLT inhibitor phlorizin [26]. As the use of SGLT2 inhibitors is rapidly increasing in the treatment of T2D patients, their potential protective effect regarding the development of diabetic retinopathy through direct actions on retinal pericytes needs to be further investigated in clinical studies.

In conclusion, our pilot study suggests an important role of *SLC5A2* polymorphisms in the physiologic process of glucose reabsorption in kidneys in T2D patients. Furthermore, we report for the first time a biologically plausible association between *SLC5A2* polymorphism and diabetic retinopathy. Further studies are needed to investigate if genetic variability of SGLT2 transporters influences treatment outcome with the novel class of antidiabetic drugs that inhibit SGLT2.

*Acknowledgements*. We wish to acknowledge the clinical nurse Mrs. Nevenka Kiri~ for her support with clinical part of the study and Mrs. Savica Soldat, BSc for her expert technical assistance.

The preliminary data of this study were presented by Klen et al. as conference abstracts published in the American Diabetes Association’s 76th Scientific Sessions Abstract Book (http://diabetes.diabetesjournals.org/content/diabetes/65/Supplement_1/A595.full.pdf). The preliminary data were also accepted for short presentation at the 41st FEBS Congress, Molecular and Systems Biology for a Better Life, Ephesus/Kuşadasi, Turkey, September 3 8, 2016. Although the congress was canceled, the abstract was published in the FEBS Journal, Volume 283 Supplement 1, September 2016 (https://febs.onlinelibrary.wiley.com/doi/epdf/10.1111/febs.13805).

*Funding*. This work was supported by the Slovenian Research Agency [grant number P1-0170].

*Disclosure*. Prof. Dolzan reports grant P1-0170 from Slovenian Research Agency; other authors have nothing to disclose.

## Conflict of interest statement

The authors stated that they have no conflicts of interest regarding the publication of this article.

## List of abbreviations

CI, confidence interval; HbA1c, glycated hemoglobin; ICD, ischemic cerebral disease; MI, myocardial infarction; N, number of patients; OR, odds ratio; PAOD, peripheral arterial occlusive disease; SGLT2, sodium-glucose cotransporter; SNP, single nucleotide polymorphism; T2D, type 2 diabetes; TmG, transport maximum for glucose reabsorption
